# A Detection Transformer-Based Intelligent Identification Method for Multiple Types of Road Traffic Safety Facilities

**DOI:** 10.3390/s24103252

**Published:** 2024-05-20

**Authors:** Lingxin Lu, Hui Wang, Yan Wan, Feifei Xu

**Affiliations:** 1 College of Artificial Intelligence, Southwest University, Chongqing 400715, China; lareinalu607@163.com; 2 Key Laboratory of New Technology for Construction of Cities in Mountain Area, Ministry of Education, School of Civil Engineering, Chongqing University, Chongqing 400045, China; 3 School of Civil and Transportation Engineering, Ningbo University of Technology, Ningbo 315211, China; wanyan@nbut.edu.cn (Y.W.); xufeifei@nbut.edu.cn (F.X.)

**Keywords:** road traffic safety facility, intelligent identification, detection transformer, DINO, Yolov7

## Abstract

Road traffic safety facilities (TSFs) are of significant importance in the management and maintenance of traffic safety. The complexity and variety of TSFs make it challenging to detect them manually, which renders the work unsustainable. To achieve the objective of automatic TSF detection, a target detection dataset, designated TSF-CQU (TSF data collected by Chongqing University), was constructed based on images collected by a car recorder. This dataset comprises six types of TSFs and 8410 instance samples. A detection transformer with an improved denoising anchor box (DINO) was selected to construct a model that would be suitable for this scenario. For comparison purposes, Faster R-CNN (Region Convolutional Neural Network) and Yolov7 (You Only Look Once version 7) were employed. The DINO model demonstrated the highest performance on the TSF-CQU dataset, with a mean average precision (*mAP*) of 82.2%. All of the average precision (*AP*) values exceeded 0.8, except for streetlights (*AP* = 0.77) and rods (*AP* = 0.648). The DINO model exhibits minimal instances of erroneous recognition, which substantiates the efficacy of the contrastive denoising training approach. The DINO model rarely makes misjudgments, but a few missed detection.

## 1. Introduction

A traffic safety system should include functions for traffic management, safety protection, traffic guidance, protective isolations, and antiglare [1]. The Road Traffic Safety Facility (TSF) plays a crucial role in traffic management and safety. Facility management is highly dependent on manual labor due to the complexity and diversity of TSFs. Intelligent driving requires higher TSF management due to the timeliness and accuracy of traffic information acquisition. However, unclear management responsibilities and frequent mistakes in related facility account information have led to poor management of road facility assets. Additionally, unforeseen events such as weather conditions and accidents can cause damage or loss of TSFs. The problem with TSFs can result in unforeseen safety issues, as evidenced by numerous news reports of serious traffic accidents. Despite this, TSFs have not received much attention due to their low asset values compared to infrastructure.

**Figure 1:**
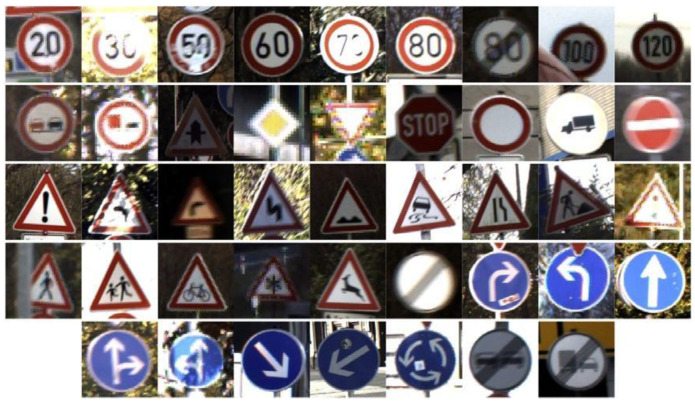
Samples of the GTSRB dataset.

**Figure 2:**
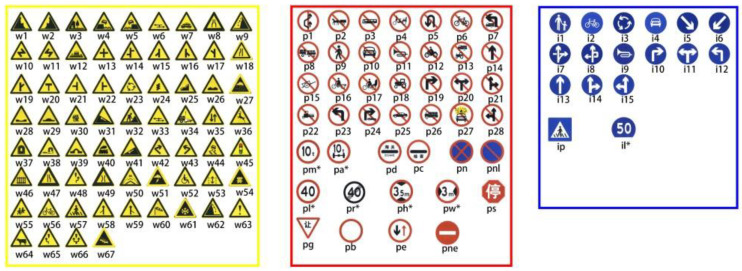
Samples from the TT100K dataset.

**Figure 3:**
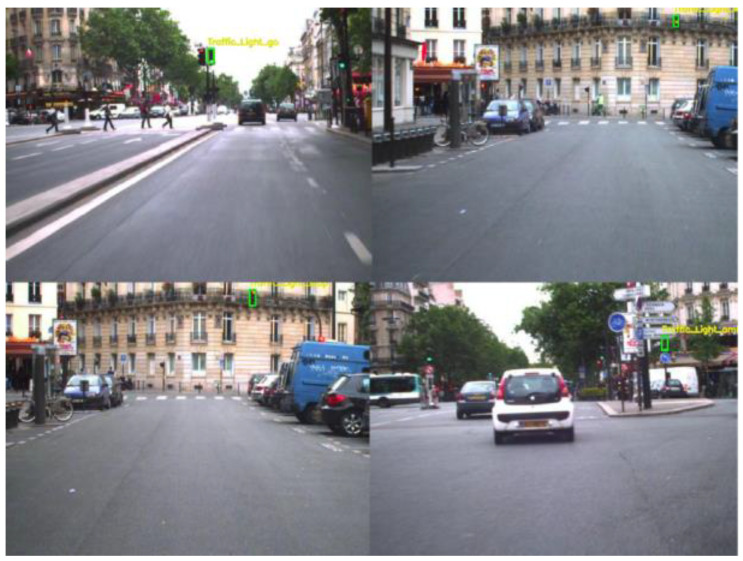
Samples of the LaRa dataset. Note: Green boxes are street light targets.

**Figure 4:**
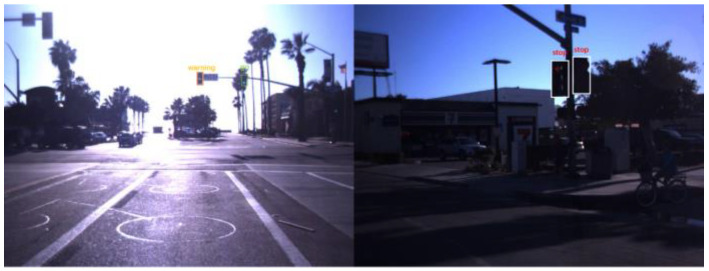
Samples from the LISA dataset.

Current TSF detection algorithms primarily consist of traffic sign recognition (TSR) and traffic light recognition (TLR) [2,3,4,5,6,7]. These algorithms are based on target detection and intelligent recognition of a single category of objects and are applied in automated driving embedded in the advanced driver assistance system. The most commonly used datasets for TSR are the German Traffic Sign Recognition Benchmark (GTSRB) [8] and the Tsing-Hua-Tencent Traffic Sign Dataset (TT100K) [9]. The GTSRB dataset comprises 43 classes of traffic signs, as illustrated in Figure 1. Meanwhile, the TT100k dataset has 221 classes, with only 128 classes labeled, as shown in Figure 2. Commonly used datasets in TLR include the Bosch small traffic lights dataset (BSTLD) [10], the French traffic light dataset (LaRa), and the California traffic lights dataset (LISA) [11]. The LaRa dataset (Figure 3) contains four categories and the LISA dataset (Figure 4) contains three categories, based on differences in traffic information such as stop and go.

As seen in Figure 1, Figure 2, Figure 3 and Figure 4, the existing TSR and TLR datasets are useless for facility classification, which represent only two types of TSFs. However, the recognition algorithms used may have some value.

Arcos-García Á et al. proposed a deep learning method to achieve fine-grained recognition of traffic signs; the recognition rate reached an accuracy of 99.71% in GTSRB, outperforming previous state-of-the-art methods [12]. Chen et al. used Yolov4 and Yolov5 as inspiration to propose an efficient method for TSR with low perception, which achieved significant performance improvements on the dataset TT100K [13]. Wang Q et al. addressed the problems of Yolov4 being insensitive to small targets and low detection accuracy in red light detection, using a shallow feature enhancement mechanism and a bounding box uncertainty prediction mechanism to improve, reaching 97.58% and 95.85% in AUC; 82.15% and 79.97% in accuracy on the LISA and LaRa datasets [14]. The enhanced YOLOv4-Tiny algorithm, which has been integrated with K-means clustering, has demonstrated an improvement in accuracy of 5.73% and a 7.29% enhancement in precision when tested on the TT100K dataset [15]. Pon A et al. developed a joint detection model for the gap research point that no public dataset contains both traffic light and traffic sign labels, based on the BSTLD and TT100k datasets. Real-time detection of traffic lights and signs can be achieved, but there is some performance loss compared to training on only one of the two datasets, with an average precision loss of 18% [16]. Both TSR and TLR are based on image data from the perspective of a forward-looking camera, similar to the TSF detection data. However, research for TSR and TLR usually focuses on real-time, high-precision detection to provide a reference for the automatic driving system. This kind of target detection fails to obtain classification information for facility asset management.

The recognition of TSFs is primarily based on the relevant account information generated during installation or post-manual statistical surveys, which can be time-consuming. Therefore, the application of intelligent recognition methods could effectively and efficiently detect TSFs. Currently, TSFs have not been used comprehensively in the scope of management or as a research identification object. Recognition of TSR and TLR is primarily used in autonomous driving, while facility management involves some differences. In autonomous driving, more attention is given to identifying auxiliary information such as traffic signals, traffic signs, and road markings. Additionally, different performance requirements are pursued, with an emphasis on real-time and accurate recognition of traffic-related information. In road asset management, identifying facilities with multiple categories and attributes is a significant challenge, making it a multi-target detection task.

In addition to studies on TSR and TLR, other fields of road detection have included TSFs as recognition targets. For instance, Ning Z et al. [17] proposed a YOLOv7-RDD model to recognize pavement distress, including the marking loss category, with an AP higher than 85%. Researchers have utilized various forms of data to detect road traffic facilities. Thanh Ha et al. [18] proposed a method for automatically detecting and classifying pole-shaped road facilities in the highway environment using MLS (Least Squares) point cloud data. The method employs a set of knowledge-based rules based on height features and geometric shapes to categorize detected road poles into different types of roadway facilities. The method was evaluated using two test points from a point cloud in a highway environment located in Bangkok, Thailand. It achieved an average F1 score of 94.3% and accurately detected and labeled road facilities. However, it faced challenges with facilities that have large inclined poles, small markers with short trunk heights, or poles with square cross-sections. Furthermore, the equipment required for LIDAR detection technology is expensive and the processing of data can be challenging.

Although research on TSF detection is still lacking, detection models for other road infrastructures and related distress can be used as references. The CNN and YOLO family of algorithms has been used for in-road facility and pavement distress detection [17,19,20,21]. Promising multi-category object detection models for both efficiency and precision are represented by Faster-RCNN [22] and YOLO series [23]. Faster R-CNN [24] has been developed to achieve better efficiency without precision loss. YOLOv3 performed the best among the three algorithms (Faster R-CNN, YOLOv3, and YOLOv4) with a UAV detection task [25]. Lei et al. proposed a deep learning method based on a pre-trained neural network architecture using Baidu Map Street View data based on Yolov3 to achieve eight types of distress detection [26]. Ning et al. proposed a YOLOv7-based effective detection model, YOLOv7-RDD, for eight types of pavement distress and three types of in-road facilities based on low-cost front-view video data with significant accuracy and efficiency [17]. The YOLOv7-tiny model is distinguished by its leading position in terms of the number of model parameters, the amount of computation, and the accuracy, which serves to illustrate the advancement of its network structure [27]. YOLOv7 [28] has been shown to perform well in multi-target detection tasks [29,30,31,32]. In addition, a detection transformer (DETR) [33] can better handle global information in the image and does not need to use the anchor frame that needs to be set manually in traditional target detection models, making the model more concise and efficient. DN-DETR [34] introduced multiscale features to enhance the perceptual field and detection capacity for small targets based on DETR. Additionally, a dynamic feature network structure was utilized, enabling the model to adaptively select the appropriate network structure based on input images, further improving performance. DINO [35] is an improved version of DN-DETR proposed by scholars from Tsinghua University and Hong Kong University of Science and Technology in the Guangdong-Hong Kong-Macao Greater Bay Area Digital Economy Research Institute (IDEA Institute). It is claimed to be the first end-to-end transformer detector that outperforms state-of-the-art (SOTA) models on the COCO leaderboard.

In conclusion, no studies related to the TSF detection target have been reported based on our limit survey. To achieve the detection goals, a TSF dataset was built in this study based on major classifications. DN-DETR, DINO, Faster R-CNN, and YOLOv7 were chosen for the comparative study, based on the overall learning effect, the two main types of models that performed better will be chosen for further comparative analysis.

## 2. Methodology

### 2.1. YOLOv7

**Figure 5:**
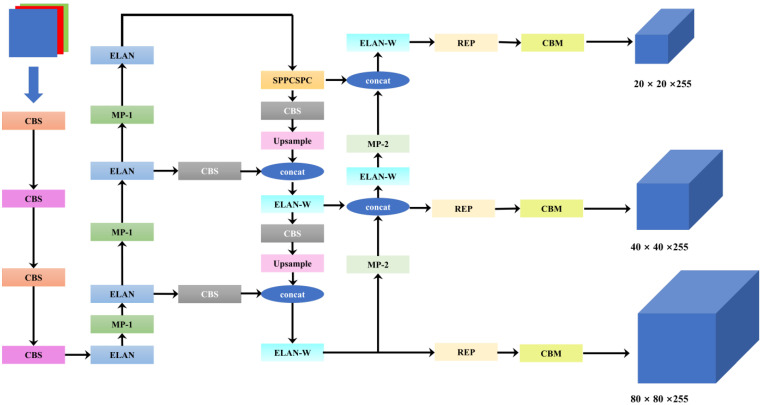
The framework of YOLOv7 (red represents a convolutional kernel of size 3 × 3 with a step of 1, purple represents a convolutional kernel of size 3 × 3 with a step of 2, and gray represents a convolutional kernel of size 1 × 1 with a step of 1).

YOLOv7 [28] proposed some architectural changes and some free bags that increased accuracy without affecting the speed of the results, it outperformed all known object detectors (including the latest YOLOv8) in terms of both performance and stability [27]. The core concept underlying the algorithm is to partition the feature map into grid cells and then to identify each cell. To illustrate, the algorithm initially processes the input image using a feature extraction network to generate a feature map with specific dimensions. This is followed by the cutting of the feature map into grid cells, each of which is responsible for detecting the targets within it. Finally, the algorithm predicts the bounding box, localization confidence, and the probability vectors of all categories of the targets contained in all the grid cells simultaneously, thus arriving at the final detection results. The structure of YOLOv7 is depicted in Figure 5.

As illustrated in Figure 5, the process can be broadly divided into the following three stages: feature extraction, feature fusion, feature parsing, and prediction. The following section provides a detailed description of each module: (1) the CBS module incorporates a convolutional layer, a batch normalization layer, and a Silu activation function layer. In the structure diagram, the different colors of the CBS module represent different sizes of convolutional kernels. (2) The ELAN module divides the input feature matrix into two branches for processing. One branch passes through a single CBS module, while the other passes through five CBS modules. Subsequently, the two branches are merged at the channel level and processed by another CBS module. (3) The MP-1 module employs a comparable branching strategy. One branch passes through the maximum pooling and CBS modules, while the other passes through two CBS modules. Afterward, the two branches are merged at the channel level. (4) The SPPCSP module also divides the input feature matrix into two branches of processing, where one branch passes through three CBS modules and three different sizes of pooling operations (5 × 5, 9 × 9, and 13 × 13) and splicing, and then passes through two CBS modules. The other branch passes through only one CBS module, after which the two are fused at the channel level. (5) The UPSample module is responsible for performing the upsampling operation for bilinear interpolation. (6) The ELAN-W module is structurally analogous to the ELAN module, with the principal distinction being in the second branch, where the number of channels in the fusion of channels converging after a single CBS module differs. The MP-2 module is analogous. The MP-1 module differs from the MP-2 module in that it has a different number of channels. The CBM module, on the other hand, consists of a convolutional layer, a batch normalization layer, and a sigmoid activation function layer. (7) The final outputs contain large-sized, medium-sized, and small-sized detection frames, which are 20 × 20 × 255, 40 × 40 × 255, and 80 × 80 × 255, respectively.

YOLOv7 divides the feature maps of the input images into grids of different sizes and achieves the fusion of multi-scale features through mechanisms such as the Feature Pyramid Network (FPN) [36], which enables the model to learn more comprehensive scale information and achieve more comprehensive target prediction. Furthermore, the novel contributions of YOLOv7 include an efficient aggregation network, model scaling, reparameterized convolution, auxiliary detection header, and dynamic label assignment. Collectively, these innovations position YOLOv7 as a leading model in the field of target detection, enabling it to efficiently perform target detection in a diverse range of complex scenarios.

### 2.2. Faster-RCNN

**Figure 6:**
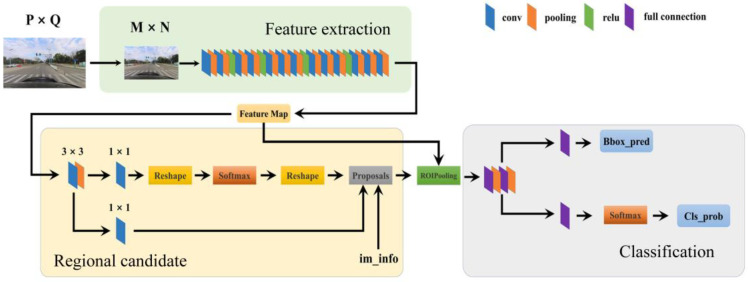
The framework of Faster R-CNN.

Among the various enhanced algorithms based on R-CNN, Faster R-CNN [24] is regarded as the most effective approach for target detection. Figure 6 illustrates the structure of the Faster R-CNN network based on the VGG16 model.

As shown in Figure 6, this network comprises the following three principal stages: feature extraction, region candidate network, and classification. The following is an example of an input image of any size to introduce the computational process of Faster R-CNN in detail. 

Assuming that the size of the input image is P × Q (P and Q are arbitrary), it will be scaled to a fixed size M × N before being fed into the feature extraction module. The feature extraction network uses VGG16, which contains 13 convolutional layers, 13 Relu activation layers, and 4 pooling layers. Subsequently, the extracted features are fed into the region proposal network (RPN), which first undergoes 3 × 3 convolution and then is divided into two lines. The upper line determines whether the anchor frames are positively or negatively correlated through a softmax classifier, while the lower line is used to compute the bounding box regression offsets for the anchor frames to obtain the accurate candidate. The RPN then obtains the precise candidate frames by filtering the anchor frames. The RPN filters out the anchor frames with the highest classification confidence from the preset anchor frames and determines these anchor frames as candidate frames. The im_info layer contains information about the size and scaling of the input image, as well as the size of the feature map and the corresponding scaling factor. In the Proposals layer, positively correlated anchor frames and anchor frame offsets are combined, while regions that are too small and out of bounds are eliminated in conjunction with im_info. The ROI pooling layer then uses candidate frame proposals and the feature map to obtain the feature representation of the region of interest after pooling. Finally, the system enters the classification stage. This involves a fully connected layer and a softmax operation for classification, as well as a bounding box regression operation to obtain the inference results.

### 2.3. DN-DETR and DINO

#### 2.3.1. Introduction of the DINO Model

**Figure 7:**
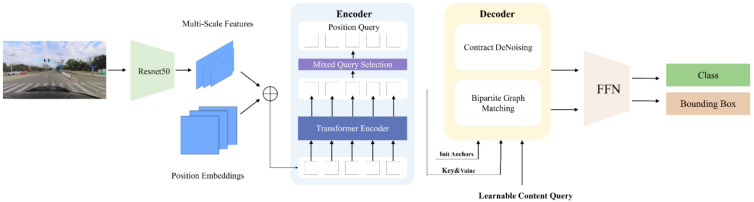
The pipeline of DINO.

DINO [35] has the following three main improvements over DN-DETR: Contrastive DeNoising (CDN) approach training, Mixed Query Selection (mixed QS), and the Look-Forward Twice (LFT) mechanism. The denoising training of DN-DETR introduces noisy samples for learning. DINO represents an end-to-end architecture comprising a backbone, a multi-layer transformer encoder, a multi-layer transformer decoder, and multiple prediction heads. The pipeline of DINO is illustrated in Figure 7.

It can be seen in Figure 7 that, given an image, we extract multi-scale features with backbones ResNet50 [37], and then feed them into the transformer encoder with corresponding positional embeddings. Following the enhancement of features with the encoder layers, a novel mixed query selection strategy is proposed for the initialization of anchors as positional queries for the decoder. It should be noted that this strategy does not initialize content queries but rather leaves them learnable.

The multiscale features are fed to the transformer encoder for feature enhancement together with the corresponding position embeddings. The deformable attention mechanism proposed in deformable DETR [38] is used in the encoder and the cross-attention part between the encoder and the decoder to combine the output of the features from the encoder and update the query layer by layer. The mixed QS module enhances the position information using top-K features after the encoder. The decoder is divided into the following two parts: (1) CDN is a contrast denoising training module that learns by introducing positive and negative samples with noise; (2) the Bipartite Graph Matching part correlates the output prediction with the real target in the input image to obtain the accurate target detection result. The output sequence of the decoder is passed through the Feed-Forward Network [39] to generate the predictions of the final category and the bounding box predictions. 

An input image and multiple regions of interest (ROIs) are input into a fully convolutional network. Each ROI is pooled into a fixed-size feature map and then mapped to a feature vector by fully connected layers. The network has two output vectors for per ROI, including softmax probabilities and per-class bounding-box regression offsets. The architecture is trained end-to-end with a multi-task loss [40]. 

#### 2.3.2. Contrastive DeNoising Approach

If the square center is identified as a ground truth (GT) box, points situated within the inner square are regarded as a positive example, whereas points situated between the inner square and the outer square are viewed as negative examples. The use of GT boxes is more beneficial in improving performance. 

**Figure 8:**
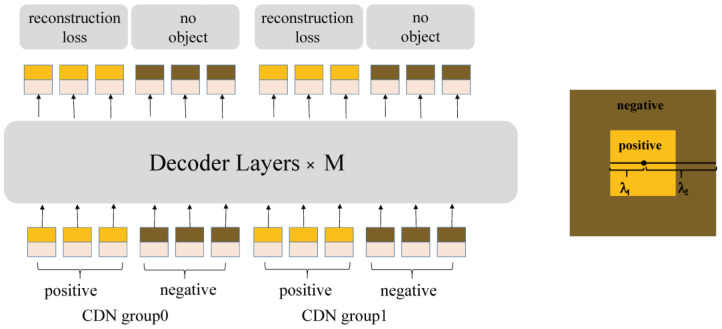
The structure of the CDN group [35].

As illustrated in Figure 8, each CDN group is comprised of a set of positive queries and negative queries. If an image contains n GT boxes, a CDN group will have 2 × n queries, with each GT box generating a positive and a negative query. Similarly to DN-DETR, multiple CDN groups are employed to enhance the efficacy of the model.

#### 2.3.3. Mixed Query Selection

**Figure 9:**
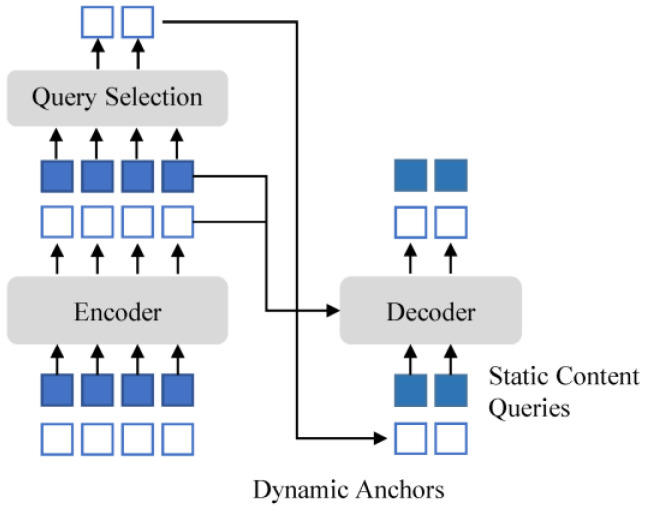
The structure of the mixed QS [35].

DINO proposes using a mixed QS module to improve the performance of the decoder query vector; the structure of the mixed QS is shown in Figure 9.

Figure 9 illustrates that anchor boxes are initialized solely with the positional information of the selected top-K features, while content queries remain static. In Deformable DETR [38], the gradient information is used to refine target frame generation during training, which has been shown to improve detection results. The selected features serve as preliminary content features, potentially encompassing multiple objects or merely a portion of an object. The mixed QS approach enhances the positional queries with the top-K-selected features while preserving the learnability of the content queries. This enables the model to utilize more comprehensive content features by utilizing better positional information.

#### 2.3.4. Look-Forward Twice Mechanism

Given an input box *b_i_*_−1_ for the *i_th_* layer, the final prediction box can be obtained using the following equations:

(1) $$\Delta b_{i}={Layer}_{i}\left(b_{i-1}\right),\;b_{i}^{\prime }=Update\left(b_{i-1},\Delta b_{i}\right),\;b_{i}=Detach\left(b_{i}^{\prime }\right),\;b_{i}^{\left(predict\right)}=Update\left(b_{i-1}^{\prime },\Delta b_{i}\right)$$

where $b_{i}^{\prime }$ is the undetached version of *b_i_* and update is a function that refines the box by the predicted box offset $\Delta b_{i}$. The update method is the same as in Deformable DETR [38].

## 3. Experiment

### 3.1. TSF-CQU Dataset

The TSF-CQU dataset was created using images captured by a low-cost vehicle recorder on municipal roads in Shanghai, Chongqing, and Ningbo. The dataset contains 1437 images, which are divided into a training set, a validation set, and a test set in a ratio of 0.85: 0.1: 0.05. Specifically, there are 1222 images in the training set, 142 images in the validation set, and 73 images in the test set. The dataset is labeled with six main categories of detection targets, as presented in Table 1. The total instance sample number is 8410. Specifically, there are 7244 instance samples in the training set, 777 instance samples in the validation set, and 389 instance samples in the test set.

**Figure 10:**
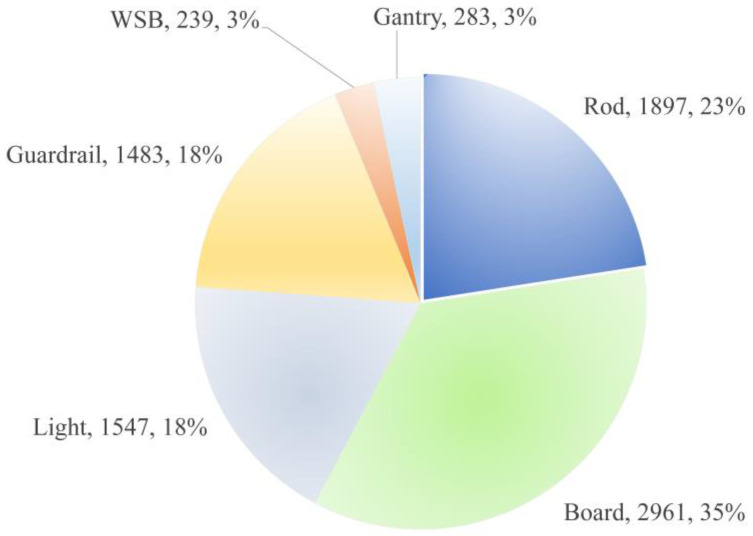
Data distribution of the TSF-CQU dataset.

**Figure 11:**
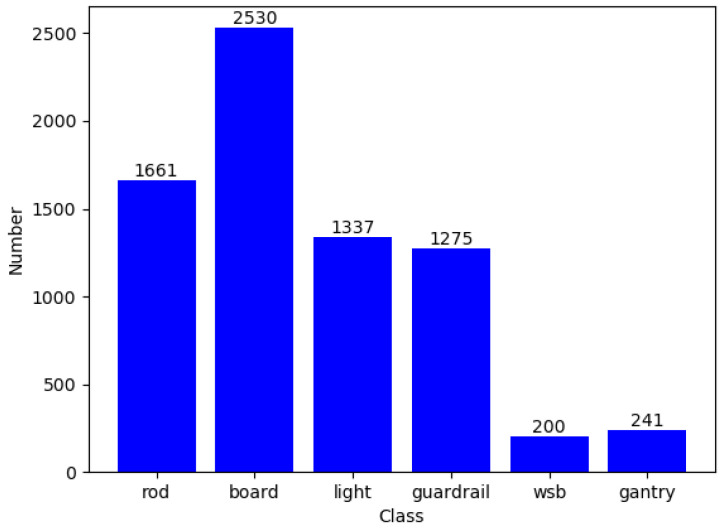
Label numbers of the training dataset.

The data distribution of the dataset is illustrated in Figure 10, and the label numbers of the training dataset are illustrated in Figure 11.

As illustrated in Figure 10 and Figure 11, the numbers of WSBs and gantries are relatively limited, while the numbers of other targets all exceed 1000 for the training dataset.

### 3.2. Evaluation Metrics

Evaluation metrics including *Precision*, *Recall*, average precision (*AP*), and mean average precision (*mAP*) were used, which are calculated by Equations (2)–(5).

(2) $$Precision=\frac{TP}{TP+FP}$$

(3) $$Recall=\frac{TP}{TP+FN}$$

(4) $$AP=\int_{0}^{1}PdR$$

(5) $$mAP=\frac{1}{N}\sum_{i=1}^{N}AP_{i}$$

where *TP* denotes true positive case numbers and *FP* denotes false positive case numbers, *Recall* is the percentage of all true targets that are detected, *AP* is the area under the Precision–Recall curve, which measures the performance of the model in a category, and *mAP* is the mean *AP* values of all categories with an intersection over union (*IoU*) threshold of 50%.

In addition, the parameter number (params) and operation volume (Giga Floating-point Operations, GFLOPs), processing frames per second (FPS), training time, and graphics memory used for training are used as the efficiency evaluation metrics of the efficiency of the final model.

### 3.3. Experimental Setup and Model Training

The experimental equipment uses an NVIDIA GeForce RTX 3090 Ti graphics card for training, which has 10,496 CUDA cores and 24 GB GDDR6X memory. The time of each epoch operation is about 140 s, which can realize the stable operation of this algorithm.

The AdamW optimizer [41] with a learning rate of 0.0001 and a weight decay coefficient of 0.0001 was used to train the network model. In the training phase, 2 real images were used as input for each iteration, and 100 epochs were trained for the experiments. The backbone network is RestNet50 and the learning rate scheduler is OneCycleLR [42] and the main idea is to use a “cycle” to adjust the learning rate during the training process. This cycle is divided into two phases; the first phase has a gradually increasing learning rate and the second phase has a gradually decreasing learning rate. The main parameters in OneCycleLR are as follows: the maximum value of the learning rate (*max_lr*) is set to 0.0001, the number of steps in each training cycle (steps_per_epoch), and the proportion of time required for the learning rate to increase to its maximum value (*pct_start*) are set to 0.2. 

The algorithm uses several loss functions to train the model, including focal loss [43] and box regression loss (BRL). Focal loss addresses the issue of category imbalance in classification problems by reducing the weights of easily classified samples and introducing an adjustable parameter gamma. This allows for more weight to be given to difficult-to-classify samples, enhancing the focus on rare categories. The Box Regression Loss (BRL) is utilized to evaluate the accuracy of the model’s prediction of the target box location. It is calculated by combining the Smooth L1 loss function and the GIoU loss function [40]. The total loss function is a weighted sum of the classification loss and the box regression loss.

### 3.4. Ablation Experiment of DINO Components

To analyze the effect of relevant components on the recognition effect, we conducted component-specific ablation experiments. The selected components included Contrastive DeNoising (CDN) or DeNoising (DN), Mixed Query Selection (mixed QS), and Look Forward Twice (LFT). The ablation experiments included the following component combination settings: DINO (CDN + mixed QS + LFT), DQL (DN + mixed QS + LFT), and DQ (DN + mixed QS).

## 4. Results and Discussion

### 4.1. The Training Process of DINO

**Figure 12:**
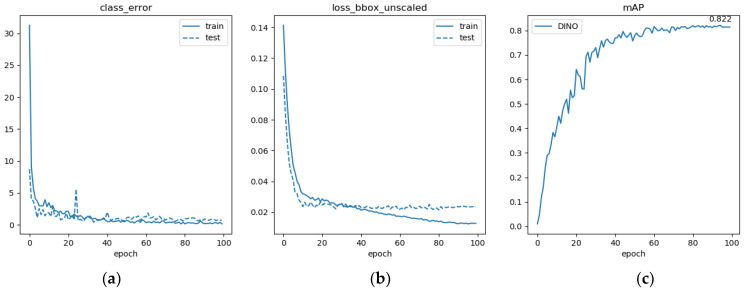
Loss curves and accuracy plots of the DINO training results. (**a**) Classification error curves, (**b**) the unscaled bounding-box regression loss curves, (**c**) mean average precision curve.

Due to the specificity in error measures of DINO, analysis of its training process was carried out. Class_error curves indicate the metric of the model’s prediction error in the classification task and are derived by calculating the classification error rate for all objects. Loss_bbox_unscaled is the unscaled bounding-box regression loss, which is not combined with other sub-losses in a weighted manner. The bounding box regression loss is used to measure the error of the model in predicting object positions, and it uses a combination of smoothed L1 loss and GIoU loss. The loss and accuracy curves in the model training results are shown in Figure 12.

Figure 12 shows that the classification error rate decreases from over 30% to almost zero during the 100 epochs of training. However, the classification loss of the validation set is higher and more volatile than that of the training set. The loss_bbox_unscaled decreases and converges during the training process, dropping below 0.02 in the test set at the end of the training, while remaining relatively high (above 0.02) in the validation set. The difference in loss between the validation set and the training set may be attributed to variations in the data distribution or overfitting during training. The mean average precision (*mAP*) consistently improves during training, stabilizing at 80% after 60 epochs, with a maximum of 82.2%.

### 4.2. Comparison of Models

#### 4.2.1. Training Results

**Figure 13:**
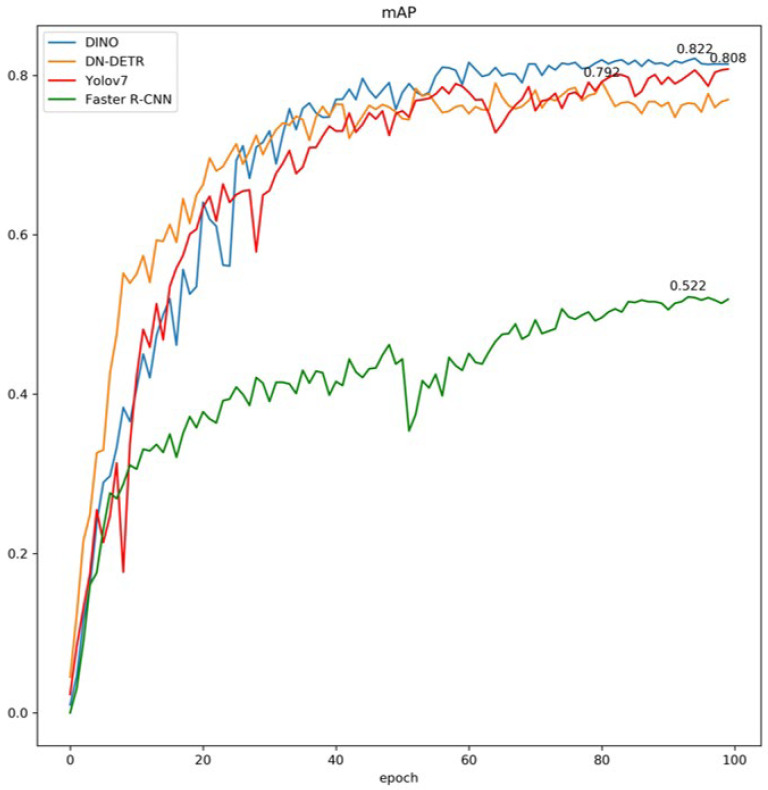
Comparison of the predicted results.

Faster R-CNN, Yolov7, DN-DETR, and DINO algorithms were selected for comparison experiments, and the training results are shown in Figure 13.

Figure 13 shows the *mAP* trend during the iterative process, with the highest *mAP* value of the corresponding model marked on the curve. DINO achieved the highest *mAP* of 82.2%, followed by Yolov7, and Faster R-CNN had the lowest performance with the highest *mAP* of only 53.4%. Both DINO and YOLOv7 exhibit high fast learning abilities in the initial period and achieve an *mAP* of around 60% with fluctuations up and down at 20 epochs. DINO exhibits better training effects in the later period and outperforms YOLOv7. DINO has significant advantages over Faster R-CNN and shows superiority compared to YOLOv7 and DN-DETR, which is reported to be the best-performing algorithm in the YOLO series.

#### 4.2.2. Comparison of Detection Precision of DINO and Yolov7 for Each Category

**Figure 14:**
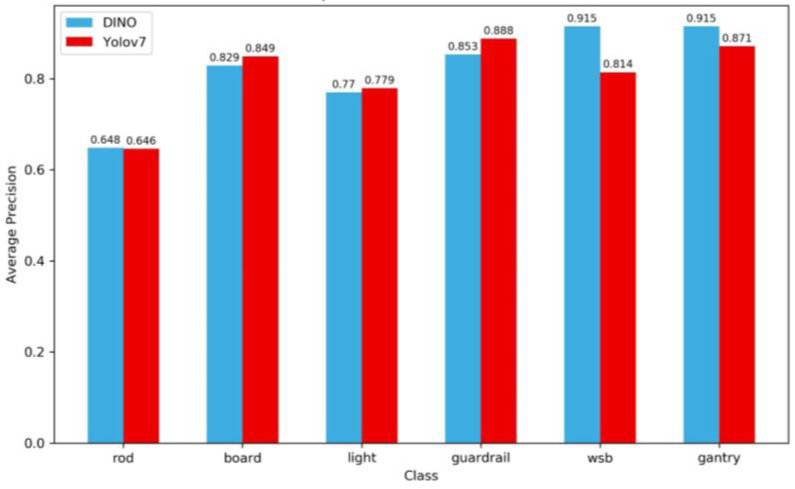
Comparison of DINO and Yolov7 for each category.

The *AP* values for each category are shown in Figure 14.

Figure 14 shows that the prediction accuracies of gantries and WSB are the highest by DINO, both reaching 91.5%, probably because gantries are large targets, while WSBs have prominent color and shape characteristics. Yolov7 performed best for guardrails, implying that the size of the target seems to be more important for Yolov7. The prediction accuracies (*APs*) of the traffic rods are the worst for both two models probably because there are too many styles of rods, such as single post, single-cantilever F, single-cantilever L, Y, etc. The recognition accuracies of streetlights are also poor, mainly because the targets are too small. Yolov7 performs a little better than DINO on some targets (board and lights), but it is not significant and requires further comparative analysis. It seems that YOLOv7 has an advantage for anchoring recognition of targets of different sizes. It is imperative to recognize that the relatively high detection accuracies observed for WSB and gantry may be attributed to the relatively limited sample sizes of these two types of targets. Nevertheless, parallels can be drawn with the detection accuracy of guardrails. The commonality between these three types of targets is their continuous, large-area nature, which is easily discernible to the detector.

**Figure 15:**
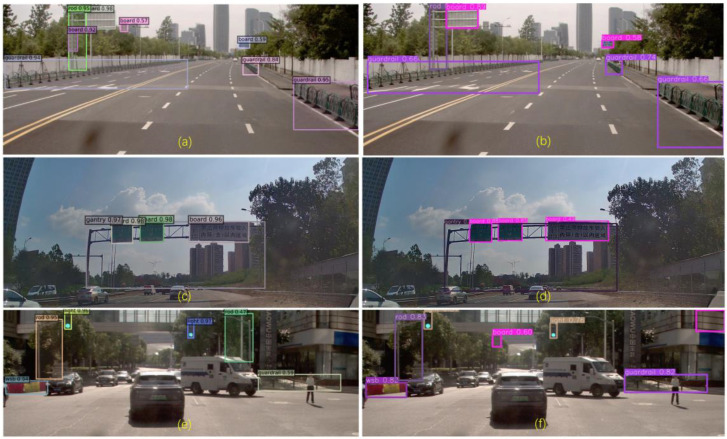
Comparison of the prediction effect of DINO (**a**,**c**,**e**) and Yolov7 (**b**,**d**,**f**).

Further sub-analysis was carried out for the two models, and the target retrieval effects are shown in Figure 15.

**Figure 16:**
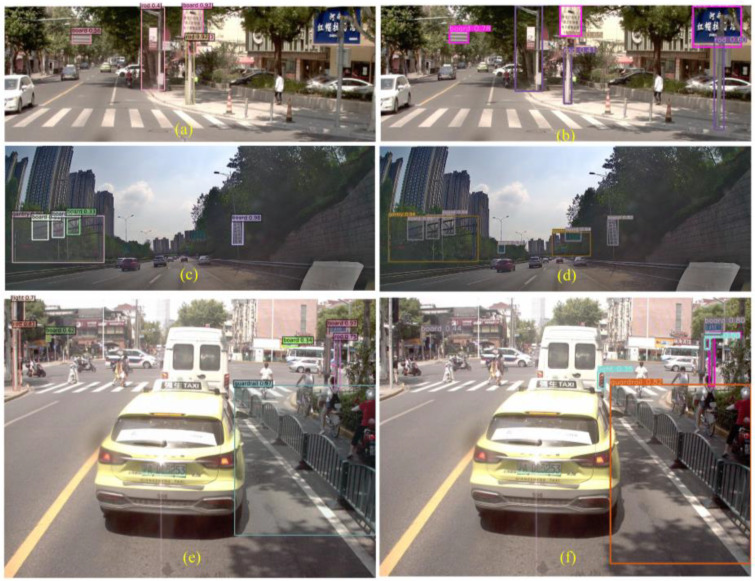
Typical error prediction samples for DINO (**a**,**c**,**e**) and Yolov7 (**b**,**d**,**f**).

**Figure 17:**
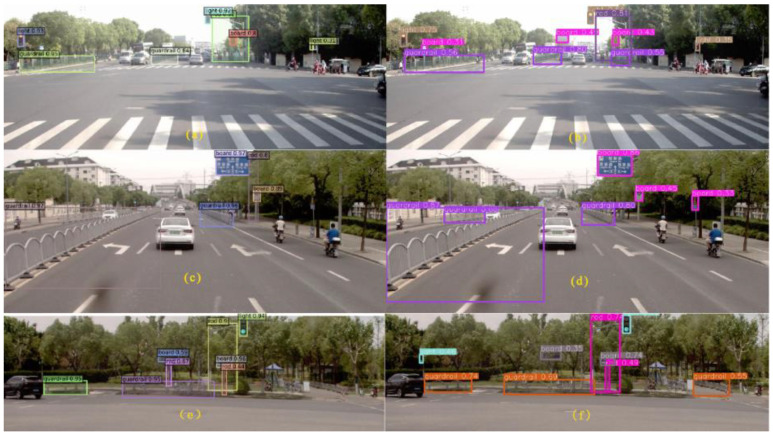
Typical error prediction samples for DINO (**a**,**c**,**e**) and Yolov7 (**b**,**d**,**f**).

Figure 15 shows that the DINO detection results are better without missing detection, and the corresponding confidence scores are higher than those of Yolov7. Yolov7 mistakenly detects the building in the upper right corner as a sign in Figure 15f, while missing the sign and traffic rod in the middle. In addition, both models do not have the problem of duplicate target detection. Representative samples of misdetections and omissions were selected to carry out further analysis, as shown in Figure 16 and Figure 17.

Figure 16 shows that Yolov7 has a serious problem with mistaken detection, while DINO has missed some targets. The front sign of the roadside store is incorrectly detected as the traffic sign board in Figure 16b, the combination of the traffic sign and the distant tall building is recognized as a gantry in Figure 16d, and the front car taillight is recognized as a traffic signal in Figure 16f. The false positive issues arise when the angle of the roadside store signage intersects with the travel angle. While DINO does not present this type of problem in this dataset, it is important to consider this issue nonetheless. Although Yolov7 has a strong self-learning ability, some errors in capturing features can mistakenly detect some other targets as our desired targets. It can be reasonably assumed that DINOs are relatively less likely to make the same types of mistakes that are commonly referred to as TN. This may be due to the CDN module’s ability to inhibit confusion. However, the DINO algorithm does not detect the sign board in Figure 16c, which may be due to the dark environment. More miss-detection samples by DINO are shown in Figure 17.

DINO does not detect the guardrail in the middle and the board in the side view on the left side in Figure 17a, the guardrail on the left side, and the board in the side view on the right side in Figure 17c, the crosswalk signal on the left side and the guardrail on the right side in Figure 17e. The samples that fail to be detected in the recognition of guardrails are usually overly compressed; this may be because the guardrails in close view usually have a larger extension but the side view does not have such an extension. The unrecognized cases of signals are usually extremely small at crosswalks, especially when the black housing of the signal is difficult to distinguish from the background in low light. In addition, certain side-view traffic signs are more difficult to recognize by DINO, which may be caused by image distortion. However, DINO can accurately identify traffic rods in Figure 17c despite the presence of tree cover, while Yolov7 missed the two rods in Figure 17d. These rods are used to support the traffic signs (boards). DINO may have learned this spatial location relationship, and this information cannot help Yolov7, which only processes once for each target identification. 

**Figure 18:**
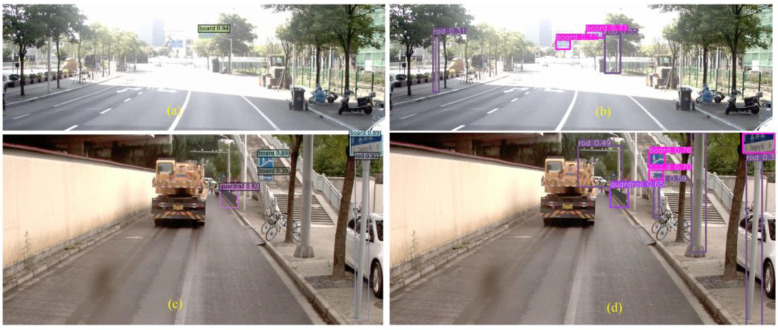
Typical rod error prediction samples for DINO (**a**,**c**) and Yolov7 (**b**,**d**).

It has been observed that DINO systems are more prone to missing detections and producing FN-type errors rather than TN-type errors. This characteristic may be disadvantageous for facility censuses. The above three types of missed recognitions may be because the DINO model processes the target as some kind of error information, which may be also caused by the CDN training approach. In addition to the three types of targets analyzed above, we will further analyze the rod targets with the lowest classification recognition *AP*. Examples are shown in Figure 18.

As illustrated in Figure 18, there are numerous instances of missed traffic rods. The majority of instances of missed traffic rods are single-post or single-cantilever types, with some variations in appearance. In these instances, Yolov7 demonstrates superior performance, with no instances of misjudgment. It appears that YOLOv7 is capable of resolving the bright and dark issues of the image. However, the detection performance of DINO for rods is not consistent across different interference backgrounds. It is evident that DINO is capable of identifying traffic rods that are partially obscured by greenery in Figure 17c, yet it fails to detect the larger one that is not entirely visible in Figure 18c.

### 4.3. Ablation Experiment Results

#### 4.3.1. Comparison of the Overall Training Results

**Figure 19:**
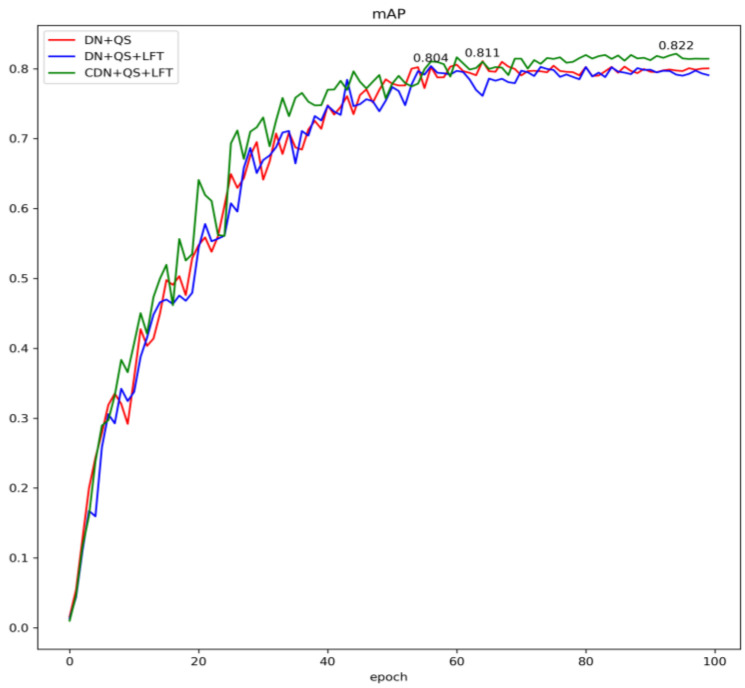
A comparison of the overall training results.

**Figure 20:**
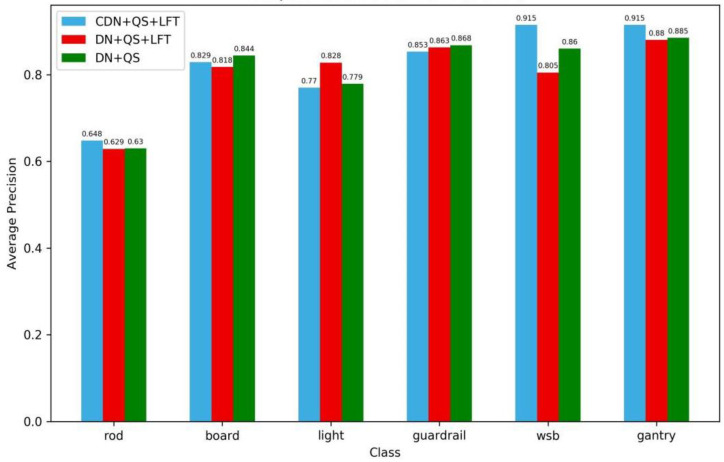
A comparison of the test results by category.

A comparison of the overall training results is shown in Figure 19, and a comparison of the test results by category is shown in Figure 20.

As seen in Figure 19, the maximum *mAP* values for DINO, DQL, and DQ are 0.822, 0.811, and 0.804, respectively. The performance of the whole *mAP* curves shows that DINO with all three components retained achieved the best results. However, the performance rankings of the three models for different categories have changed (Figure 20). 

From Figure 20, it can be seen that DINO performed poorly in detecting light and boards, DQ performed best in detecting boards, and DQL performed best in detecting lights. CDN leads to poorer recognition accuracy for light, but can effectively improve the recognition accuracies of WSB and gantry. The performance of DQ and DQL implies that LFT may play a role in accuracy enhancement for the recognition of light and guardrails, but degrade the detection performance for WSB and boards. It is worth noting that DQL (Figure 20) outperforms DINO and Yolov7 (Figure 14) in recognition of traffic lights. The diminished precision of traffic light identification resulting from the use of CDN is consistent with the capacity to diminish the occurrence of TN errors. However, the effect of is disadvantageous in the identification of small targets.

#### 4.3.2. Case Analysis

**Figure 21:**
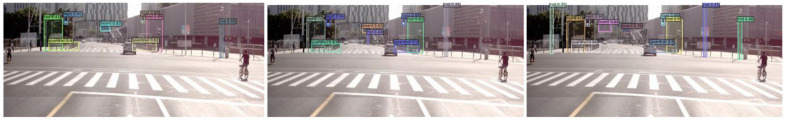
Sample 1 of recognition results (from left to right: DINO, DQL and DQ).

**Figure 22:**
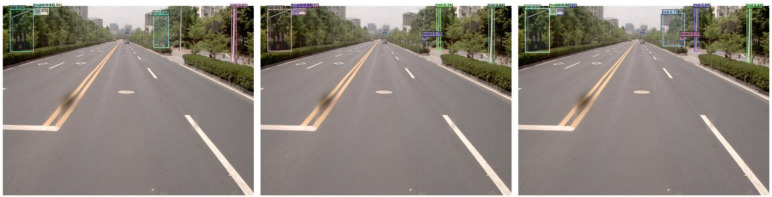
Sample 2 of recognition results (from left to right: DINO, DQL and DQ).

**Figure 23:**
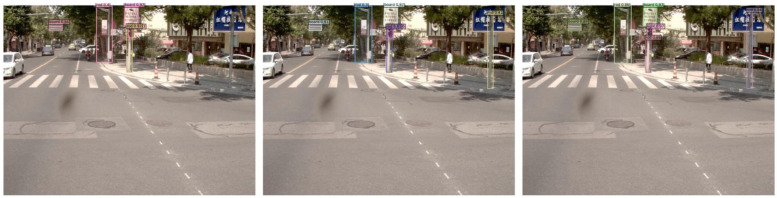
Sample 3 of recognition results (from left to right: DINO, DQL and DQ).

**Figure 24:**
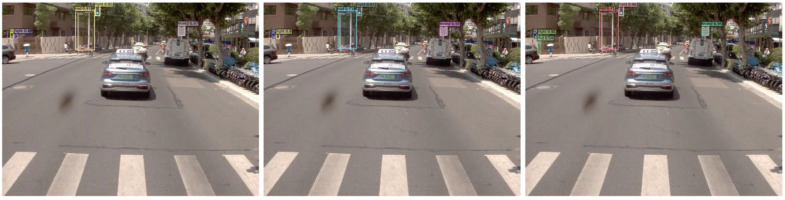
Sample 4 of recognition results (from left to right: DINO, DQL and DQ).

**Figure 25:**
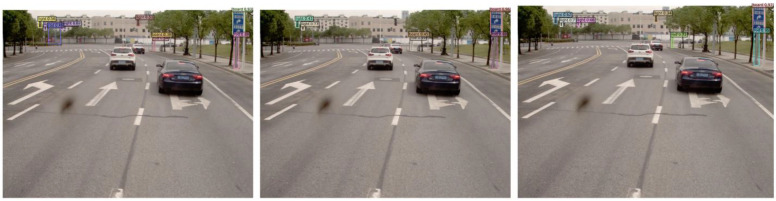
Sample 5 of recognition results (from left to right: DINO, DQL and DQ).

Case studies were developed to study the ability to recognize TSFs; the samples were selected for poorly detected rods. The detection results are shown in Figure 21, Figure 22, Figure 23, Figure 24 and Figure 25.

As seen in Figure 21, Figure 22 and Figure 23, both DQL and DQ additionally detected traffic rods compared to DINO. DQ detected the two traffic rods on the right side in Figure 21 and the rod in distant location in Figure 22, which has not been achieved by the other two models. Figure 24 shows that only DQ detected the thin rod under the streetlight on the left edge. LFT seems counterproductive for fine target detection, including the thin rod in Figure 21 and the small board in Figure 22. Although the statistics (Figure 20) show that CDN and LFT have positive effects on rod recognition, specific samples in Figure 21, Figure 22, Figure 23 and Figure 24 demonstrate the opposite conclusion. However, Figure 25 shows that DINO can detect the traffic rod of Y-type, while DQL and DQ did not complete this task, which supports the statistical conclusions.

### 4.4. Efficiency Analysis of the DINO Model for TSF Detection

The objective of this study was to achieve a comprehensive census of roadway facilities, rather than to monitor their status in real-time. However, in anticipation of future applications, we expanded the evaluation and analysis of model efficiency. The efficiency evaluation conducted for the model constructed in this study is detailed in Table 2.

Table 2 indicates that the differences between DINO, YOLO and Faster R-CNN, in terms of training time and computer graphics memory usage are within an acceptable range. The size of the model parameters is deemed to be within an acceptable range, yet the 279 GFLOPs indicate a relatively high computational complexity. Additionally, the processing speed of 24 frames per second does not meet the requirements of real-time detection.

## 5. Conclusions

With the goal of TSF surveys, DINO was chosen to design a model to effectively detect multiple TSF targets. Faster-RCNN and Yolov7 were selected in our comparative study. This study emphasizes a more comprehensive and accurate identification method of TSF assets than the intelligent recognition of traffic information that is available in the field of autonomous driving to assist in driving decision-making. The main contributions of this paper are as follows:

- Traffic safety facilities were included in the study as intelligent recognition objects from an asset management perspective, and a target detection dataset called TSF-CQU was constructed, including six types of TSFs, 1437 images, and 8420 instances.
- Using the DINO model, accurate recognition of TSFs was successfully achieved with an *mAP* of 82.2%, but the advantage over Yolov7 is not significant.
- The DINO model rarely makes misjudgments, but there is a certain degree of missed detection, mainly including traffic rods.
- CDN is not conducive to the detection of lights, and LFT is counterproductive for the detection of boards. DINO provides the most significant improvement in the detection of continuous-type large targets, such as WSBs and gantries, in comparison to DQ and DQL.

The limitation of this study is that the categorization of some of the continuous facility targets is open to question, and some of the major facility categories encompass a wide range of subcategories. Consequently, a comprehensive assessment of the categorization approach is required to determine its suitability for target detection and facility management needs. For future work, more detailed classification needs to be performed to balance the dataset distribution and improve detection precision and DINO can be improved by introducing a deformation attention mechanism, correcting the effect of the noise learning mechanism, etc. In addition, the efficiency aspects of the model need to be further investigated.

## Figures and Tables

**Table 1:** The six main categories of TSFs.

NO.	Categories	Examples
1	Traffic rod (Rod) Note: Traffic warn information in Chinese.	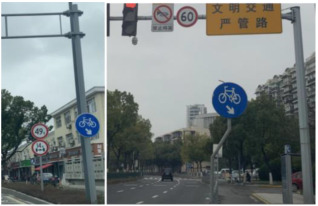
2	Traffic sign (Board) Note: Traffic warn and guide information in Chinese are shown on the board.	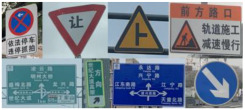
3	Light	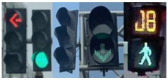
4	Guardrail	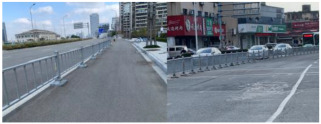
5	Water surround barrier (WSB/wsb)	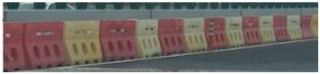
6	Gantry Note: Traffic guide information in Chinese are shown on the board.	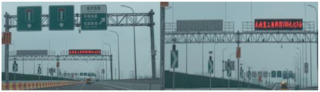

**Table 2:** The efficiency evaluations of the DINO model for TSF detection.

Models	Params	GFLOPs	FPS	Training Time of 100 Epochs	Graphics Memory Used for Training
DINO	46.6 M	279	24	9 h 35 min	9.75 G

## Data Availability

The raw data supporting the conclusions of this article will be made available by the authors upon request.
